# CT radiation dose reduction in patients with total hip arthroplasties using model-based iterative reconstruction and orthopaedic metal artefact reduction

**DOI:** 10.1007/s00256-019-03206-z

**Published:** 2019-04-24

**Authors:** Ruud H. H. Wellenberg, Jochen A. C. van Osch, Henk J. Boelhouwers, Mireille A. Edens, Geert J. Streekstra, Harmen B. Ettema, Martijn F. Boomsma

**Affiliations:** 1 Department of Radiology, Isala, Dokter van Heesweg 2, 8025 AB Zwolle, The Netherlands; 2 Department of Radiology and Nuclear Medicine, Amsterdam UMC, University of Amsterdam, Amsterdam Movement Sciences, Amsterdam, The Netherlands; 3 Department of Innovation and Science, Isala, Zwolle, The Netherlands; 4 Department of Biomedical Engineering and Physics, Amsterdam UMC, Amsterdam, The Netherlands; 5 Department of Orthopedic Surgery, Isala, Zwolle, The Netherlands

**Keywords:** Metal artifacts, Total hip arthroplasty, Metal-on-metal, CT, Radiation dose reduction, Hip prosthesis, MBIR, IMR, O-MAR

## Abstract

**Objective:**

To evaluate the impact of radiation dose reduction on image quality in patients with metal-on-metal total hip arthroplasties (THAs) using model-based iterative reconstruction (MBIR) combined with orthopaedic metal artefact reduction (O-MAR).

**Materials and methods:**

Patients with metal-on-metal THAs received a pelvic CT with a full (FD) and a reduced radiation dose (RD) with −20%, −40%, −57%, or −80% CT radiation dose respectively, when assigned to group 1, 2, 3, or 4 respectively. FD acquisitions were reconstructed with iterative reconstruction, iDose^4^. RD acquisitions were additionally reconstructed with iterative model-based reconstruction (IMR) levels 1–3 with different levels of noise suppression. CT numbers, noise and contrast-to-noise ratios were measured in muscle, fat and bladder. Subjective image quality was evaluated on seven aspects including artefacts, osseous structures, prosthetic components and soft tissues.

**Results:**

Seventy-six patients were randomly assigned to one of the four groups. While reducing radiation dose by 20%, 40%, 57%, or 80% in combination with IMR, CT numbers remained constant. Compared with iDose^4^, the noise decreased (*p* < 0.001) and contrast-to-noise ratios increased (*p* < 0.001) with IMR. O-MAR improved CT number accuracy in the bladder and reduced noise in the bladder, muscle and fat (*p* < 0.01). Subjective image quality was rated lower on RD IMR images than FD iDose^4^ images on all seven aspects (*p* < 0.05) and was not related to the applied radiation dose reduction.

**Conclusion:**

In RD IMR with O-MAR images, CT numbers remained constant, noise decreased and contrast-to-noise ratios between muscle and fat increased compared with FD iDose^4^ with O-MAR images in patients with metal-on-metal THAs. Subjective image quality reduced, regardless of the degree of radiation dose reduction.

## Introduction

In computed tomography (CT), the use of iterative reconstruction (IR) and model-based iterative reconstruction (MBIR) techniques enables a reduction in the CT radiation dose while improving image quality compared with standard filtered back-projection (FBP) [1].

Iterative reconstruction, or so-called hybrid reconstruction techniques, are blending techniques that combine IR images with FBP [2]. Some commercially available IR techniques are iDose^4^ (Philips), Safire (Siemens), ASIR (GE) and AIDR 3D (Toshiba). Philips’ MBIR algorithm iterative model-based reconstruction (IMR) is a full iterative reconstruction technique, which improves the reconstruction process by incorporating system models and photon statistics [3]. Other vendors have similar MBIR techniques named ADMIRE (Siemens), Veo (GE) and FIRST (Toshiba). With MBIR, images can be reconstructed at RDs and low noise levels. MBIR, furthermore, reduces the size of metal artefacts and allows an equal or better visibility of the bone–metal interfaces and improves the assessment of soft tissue surrounding implants compared with FBP [4, 5]. The downside of MBIR is the fact that images may appear smooth [6, 7]. More importantly, the possible loss of small details and structures could impede its use in musculoskeletal CT imaging.

In addition, metal hardware impairs the diagnostic value of CT in musculoskeletal imaging. Severe metal artefacts in the case of large-head metal-on-metal total hip arthroplasties (THAs) are mainly caused by extensive photon starvation and affect a reliable diagnosis of prosthesis-related soft-tissue and bone abnormalities. Philips’ orthopaedic metal artefact reduction algorithm, O-MAR, reduces severe metal artefacts in large implants [8–12]. Similar MAR software techniques by other vendors are iMAR (Siemens), SmartMAR (GE) and SEMAR (Toshiba). O-MAR post-processes the projection data and provides more regular attenuation profiles before image reconstruction. These more regular attenuation profiles can improve the general performance of iDose^4^ and IMR. A previous THA phantom study showed that the combined use of IMR and O-MAR enabled a radiation dose reduction of 83% where CT number accuracy, signal-to-noise ratios and contrast-to-noise ratios (CNRs) increased and noise decreased compared with the iterative reconstruction technique iDose^4^ and O-MAR [13]. However, a clinical validation study is essential to determine if the CT radiation dose can be reduced in THA patients by using IMR and O-MAR as well.

Therefore, the aim of this study was to quantitatively and qualitatively assess image quality using IMR with O-MAR while reducing CT radiation dose up to 80% in patients with large-head metal-on-metal total hip prostheses compared with full-dose (FD) iDose^4^ with O-MAR.

## Materials and methods

### Patient selection

Patients with unilateral or bilateral metal-on-metal total hip prosthesis who were scheduled for a routine 5- or 10-year follow-up CT scan for the investigation of progression or regression of pseudo tumour formation were prospectively included between October 2016 and September 2017 (Table 1). Institutional Review Board (IRB) approval was received for acquiring an additional RD CT scan in patients older than 60 years, with an explicit written informed consent for participation in this study (NL58001.075.16). Exclusion criteria were previous participation in the study, pregnancy or concomitant participation in a study in which the patient is exposed to X-rays. In total, 76 of the 120 patients who qualified to receive a 5- to 10-year follow-up CT were willing to participate in this study.

**Table 1:** Patient characteristics including age, sex, unilateral and bilateral prosthesis, location of the metal-on-metal total hip arthroplasty (*THA*), reduced dose (*RD*) and full-dose (*FD*) dose–length product (*DLP*) and CT dose index (*CTDI*)

	Group 1	Group 2	Group 3	Group 4	Total
Number of patients	20	19	18	19	76
Age (years)	72.0 ± 5.1 (61–83)	72.1 ± 5.8 (63–81)	73.6 ± 4.3 (68–85)	74.6 ± 3.6 (68–82)	73.0 ± 4.8 (61–85)
Sex (male/female)	9/11	10/9	5/13	11/8	35/41
Unilateral/bilateral	10/10	10/9	5/13	5/14	30/46
Metal-on-metal THA (left/right/both)	9/9/2	4/13/2	11/7/0	9/7/3	33/36/7
DLP FD (mGy–cm)	561.3 (446.3–839.5)	544.9 (436.4–686.2)	550.8 (454.9–699.3)	706.8 (467.8–1,007.0)	560.0 (446.3–839.5)
DLP RD (mGy–cm)	459.2 (356.8–697.8)	321.0 (257.2–408.8)	228.4 (190.3–297.7)	145.7 (101.5–267.5)	
CTDI FD (mGy)	27.2 (23.0–41.5)	24.9 (20.2–32.2)	25.1 (21.8–34.5)	29.4 (20.7–50.9)	26.5 (21.0–41.5)
CTDI RD (mGy)	22.1 (18.3–33.1)	14.2 (11.5–18.7)	10.2 (8.8–14.0)	6.2 (4.4–12.4)	

### Image acquisition and reconstruction

**Fig. 1:**
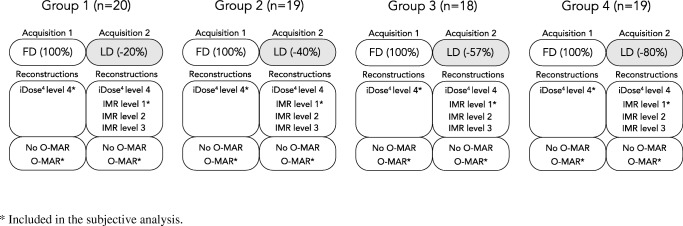
A schematic representation of the full-dose (FD) and low-dose (LD) acquisitions and reconstructions that were evaluated in all groups. Only the mAs was modified to reduce the CT radiation dose. All reconstructions were taken into account in the objective analysis where only the iDose^4^ level 4 and iterative model-based reconstruction (*IMR*) level 1 images reconstructed with orthopaedic metal artefact reduction (*O-MAR*) were subjectively analysed (*asterisk*)

Patients were scanned on an iCT 256-slice CT scanner (Philips Healthcare, Best, The Netherlands) and received a consecutive FD pelvic CT and an RD pelvic CT with 20%, 40%, 57% or 80% reduced CT radiation dose for patients in group 1, 2, 3 or 4 respectively. Patients were randomly assigned to one of the four groups (Table 1). No intravenous contrast medium was used. Scanning parameters were 140 kVp, 1.0-mm slice thickness, 0.5-mm increment, 500-mm field-of-view, 0.398 pitch and a 768 × 768 image matrix. Dose modulation and dose right index (DRI) were used to reduce the radiation dose of the RD scans based on the FD scans. Images were reconstructed with iterative reconstruction, iDose^4^ and IMR, which can be used at 7 and 3 different levels of noise suppression respectively (Philips Healthcare, Best, The Netherlands). All FD scans were reconstructed using iDose^4^ level 4, which is the middle level in terms of noise suppression. Level 4 was chosen as this is used in current clinical practice. All RD scans were reconstructed using iDose^4^ level 4 and IMR levels 1, 2 and 3 (Fig. 1). Filter type was matched for iDose^4^ and IMR reconstruction using filter D and filter SharpPlus respectively. All images were reconstructed with and without the use of O-MAR (Philips Healthcare, Best, The Netherlands).

### Quantitative analysis

**Fig. 2:**
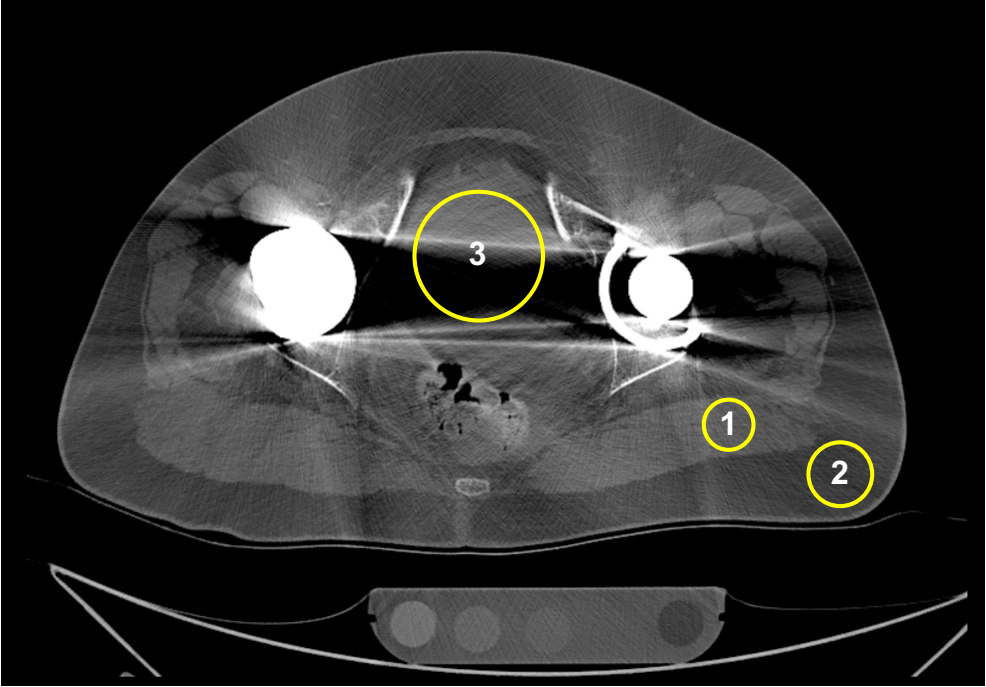
A FD acquisition reconstructed with iDose^4^ is shown. Regions of interest were placed in muscle (*1*), fat (*2*) and bladder (*3*)

A thin axial slice, containing the largest diameter of the head of the metal-on-metal prosthesis with most severe artefacts was used for the CT measurements. CT numbers, noise or standard deviation (SD) and CNRs between muscle and fat were measured in the gluteus maximus muscle, fat and bladder. CNRs were determined by dividing the absolute CT number difference between muscle and fat by the average SD of muscle and fat. Circular regions of interest (ROIs) were placed (by RW) in homogeneous areas where ROIs in muscle and fat were placed at the side with the least artefacts where the ROI in the bladder was placed within the artefact area. Size of the different ROIs was optimised for each patient as differences in anatomy preclude the use of uniform ROIs. Patient movement was limited as the FD and RD scans were acquired sequentially. However, as some patient movement could occur, FD and RD ROI templates were made for each patient scan (Fig. 2).

### Qualitative analysis

Images reconstructed with O-MAR were used for further qualitative analysis. Thin axial FD images reconstructed with iDose^4^ level 4 and RD images reconstructed with IMR level 1 were analysed (Fig. 1). IMR level 1 was chosen because quantitative results were already better than FD results in terms of noise and CNR. IMR levels 2 and 3 result in even lower noise values and higher CNR, but also increases the plastic appearance. Images were blinded and were randomly and independently evaluated on seven aspects using four-point Likert scales by two musculoskeletal radiologists with 9 years’ (MB, observer 1) and 23 years' (HB, observer 2) experience. We furthermore precluded the consecutive presentation of FD and RD images of the same patient.

Artefacts (*1*) were classified as 0: severe artefacts with a poor diagnostic quality, 1: moderate artefacts with an impaired diagnostic quality, 2: minor artefacts with a good diagnostic quality, or 3: absence of artefacts with excellent diagnostic quality. The delineation of bony structures (*2*), evaluation of bone density, destruction and sclerosis (*3*) and evaluation of prosthetic components (*4*) were classified as 0: poor evaluation with very low confidence, 1: impaired evaluation with low confidence, 2: good evaluation with medium confidence, or 3: excellent evaluation with high confidence. Evaluation and presence of pseudo tumours (*5*), evaluation of atrophy/hypertrophy and fatty infiltration of relevant hip muscles, including the obturator internus, gluteus medius, quadratus femoris/gluteus maximus and iliopsoas/tensor fasciae latae (*6*) and evaluation of the recto-uterine pouch, vesico-uterine pouch and bladder wall (*7*) were classified as 0: poor anatomic recognition and delineation with very low confidence, 1: impaired anatomic recognition and delineation with low confidence, 2: good anatomic recognition and delineation with medium confidence, or 3: excellent anatomical recognition and delineation and high confidence. Observers were able to switch between both bone window (1,600/400) and soft-tissue window (350/40). The training session consisted of a joint meeting with both radiologists where several random cases were evaluated on all seven aspects. The training cases included iDose^4^ and IMR images with different grades of pathology, artefact severity and radiation dose reduction of patients with unilateral and bilateral prostheses to provide a fair representation of the population and to elaborate on differences in scores.

### Statistical analysis

Shapiro–Wilk test and visual inspections were performed to determine whether data were normally distributed. Wilcoxon signed rank test was used to compare CT numbers, noise and CNR of the FD and RD images and O-MAR versus no O-MAR results due to relatively small sample sizes. Wilcoxon signed rank test was also used to compare subjective image quality scores by radiologists of FD and RD images. Differences in Likert scores in FD and RD images between groups were analysed using Fisher’s exact test. A significance level of 5% was used for all tests and all tests were two-tailed. SPSS software, version 24 was used.

## Results

In total 20, 19, 18 and 19 patients were included in groups 1, 2, 3 and 4 respectively (Table 1). Mean dose–length products (DLPs) decreased from 650 to 535 mGy–cm (−18%), 600 to 359 mGy–cm (−40%), 621 to 272 mGy–cm (−56%) and 725 to 173 mGy–cm (−76%) in groups 1, 2, 3 and 4 respectively.

### Quantitative analysis

**Fig. 3:**
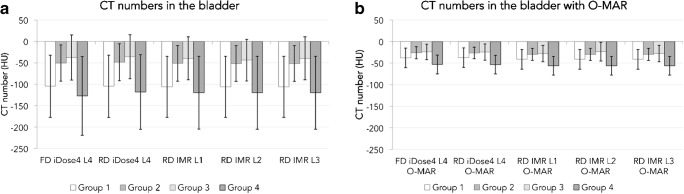
Mean CT numbers measured in the bladder **a** without and **b** with the use of O-MAR in FD and reduced dose (RD) iDose^4^ results and RD IMR levels 1, 2 and 3 results for all four groups

Orthopaedic metal artefact reduction (O-MAR) reduced noise in the bladder by 37%, 48%, 42% and 32% and increased mean CT values in the bladder (Fig. 3) with 66, 22, 14 and 66 HU in groups 1, 2, 3 and 4 respectively (*p* < 0.01). In muscle and fat, CT numbers were not statistically different in O-MAR and no O-MAR images. O-MAR furthermore decreased noise in ROIs placed in muscle and fat in RD and FD reconstructions (*p* < 0.001). O-MAR increased the CNRs between muscle and fat in FD and in RD reconstructions (*p* < 0.001).

**Fig. 4:**
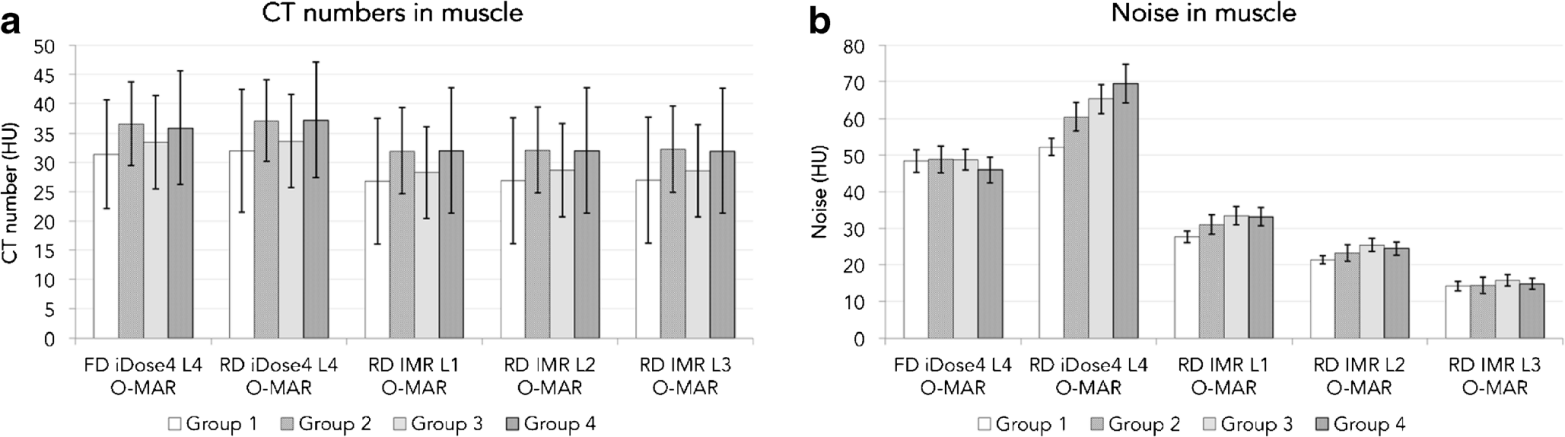
**a** Mean CT numbers and **b** noise or standard deviations measured in muscle in FD and RD iDose^4^ results and RD IMR level 1, 2 and 3 results with the use of O-MAR, for all four groups

**Fig. 5:**
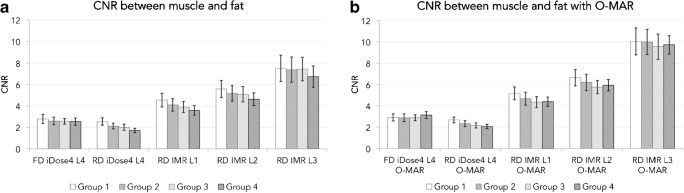
Contrast-to-noise ratio (*CNR*) between muscle and fat **a** without and **b** with the use of O-MAR

Computed tomography numbers of muscle were slightly lower in IMR 1, 2 and 3 images compared with iDose^4^ level 4 images (*p* < 0.001; Fig. 4a). Similar results were seen in fat (*p* < 0.001). When reducing the radiation dose by 20%, 40%, 57% or 80%, CNRs decreased when comparing FD iDose^4^ level 4 + O-MAR images with RD iDose^4^ level 4 + O-MAR images with 8%, 19%, 25% and 35% respectively (Fig. 5). With IMR, noise decreased (*p* < 0.001) and CNRs (*p* < 0.001) increased in RD IMR 1, 2 and 3 images compared with the FD iDose^4^ level 4 images (Figs. 4b and 5). In RD IMR level 1 with O-MAR images, CNRs between muscle and fat were on average 78%, 62%, 52% and 40% higher compared with FD iDose^4^ level 4 with O-MAR images in groups 1, 2, 3 and 4 respectively (Fig. 5).

### Subjective analysis within observers

**Fig. 6:**
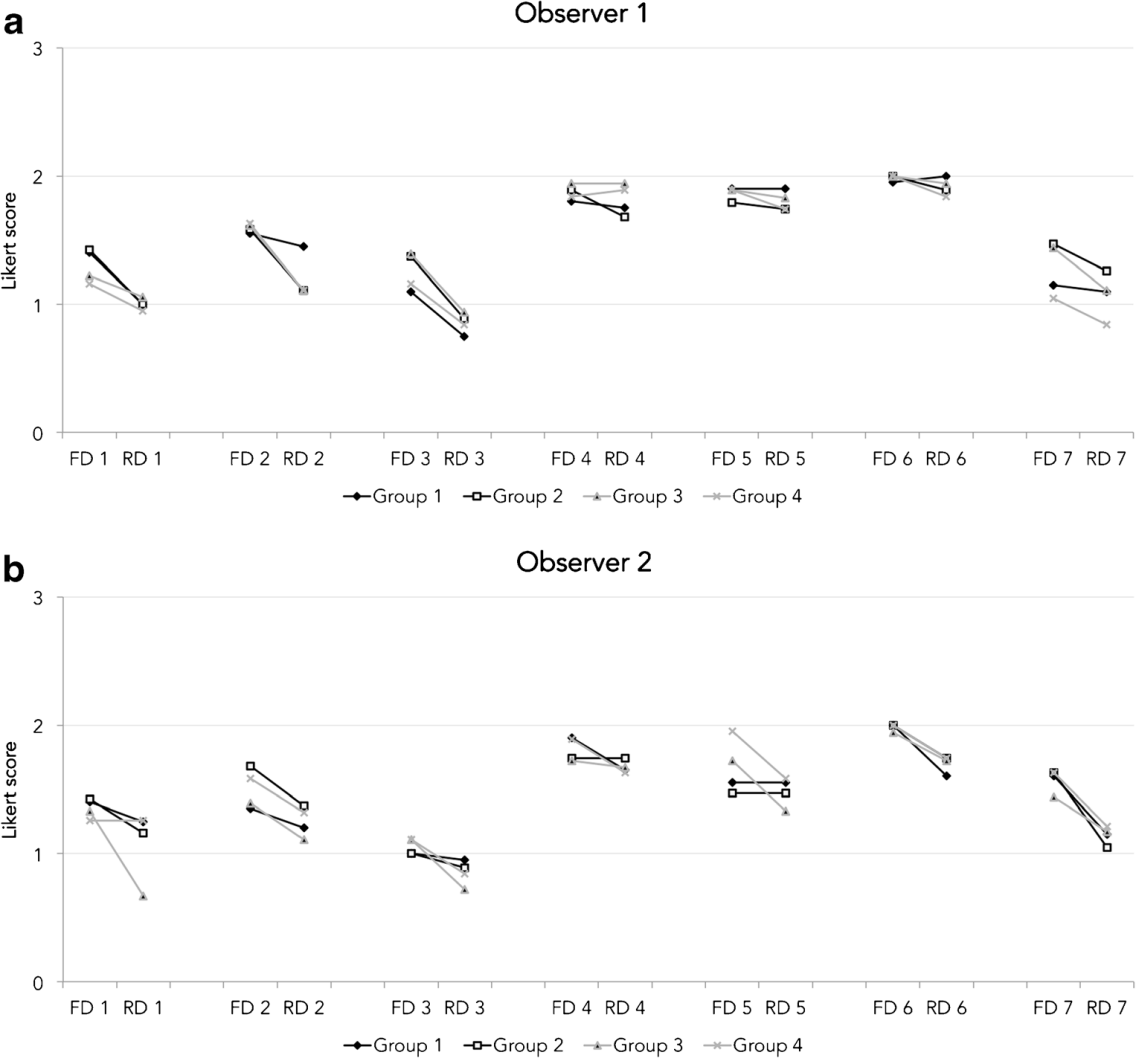
Average subjective image quality scores of all seven aspects and patients assigned to groups 1, 2, 3 and 4 for both observers. Observer 1 rated RD IMR images inferior to FD iDose^4^ images on aspects 1, 2, 3 and 7. Observer 2 rated the RD IMR images inferior to FD iDose^4^ images on all seven aspects

For observer 1, mean subjective image quality scores were lower in RD IMR with O-MAR compared with FD iDose^4^ with O-MAR regarding the following aspects: artefacts and diagnostic quality (*p* < 0.001), delineation of bony structures (*p* < 0.001), evaluation of bone density, destruction and sclerosis (*p* < 0.001) and evaluation of the recto-uterine pouch, the vesico-uterine pouch and the bladder wall (*p* < 0.01). For observer 2, average subjective image quality scores in RD IMR with O-MAR were lower compared with FD iDose^4^ with O-MAR on all seven aspects: 1 (*p* < 0.01), 2 (*p* < 0.01), 3 (*p* < 0.01), 4 (*p* < 0.05), 5 (*p* < 0.05), 6 (*p* < 0.001) and 7 (*p* < 0.001; Fig. 6).

Averaging the results of both observers resulted in overall lower image quality scores on all seven aspects: 1 (*p* < 0.001), 2 (*p* < 0.001), 3 (*p* < 0.001), 4 (*p* < 0.05), 5 (*p* < 0.05), 6 (*p* < 0.001) and 7 (*p* < 0.001). There was no statistically significant difference in subjective image quality scores among the four groups. In RD IMR images with radiation dose reduced by 20%, 40%, 57% and 80%, mean Likert scores were on average 0.17, 0.25, 0.27 and 0.24 lower respectively compared with FD iDose^4^ images.

### Subjective analysis between observers

Subjective image quality scores were statistically different between the two observers with respect to aspects 3 (*p* < 0.05), 5 (*p* < 0.01) and 7 (*p* < 0.001) in FD iDose^4^ images and aspects 5 (*p* < 0.001) and 6 (*p* < 0.01) in FD IMR level 1 images (*p* < 0.05). Cohen’s Kappa values of 0.16 and lower were found, indicating no or poor reliability between observers for FD images and RD images.

## Discussion

Our study reveals that CT numbers remained constant, noise decreased and CNRs between muscle and fat increased in RD IMR reconstructions compared with FD iDose^4^ reconstructions for all levels of IMR while reducing CT radiation dose by 20%, 40%, 57% or 80%. By reducing metal artefacts, O-MAR reduced noise, improved CT number accuracy by bringing these values closer to expected CT numbers in the bladder and improved CNRs between muscle and fat. However, subjective image quality scores were lower in RD IMR level 1 with O-MAR images compared with FD iDose^4^ level 4 with O-MAR images, regardless of the degree of radiation dose reduction.

Large head metal-on-metal THAs composed of a cobalt chromium molybdenum alloy caused severe metal artefacts, mainly because of extensive photon starvation. These artefacts were reduced, which resulted in an improvement of CT number accuracy in the bladder and a reduction of noise. O-MAR reduced artefacts in the bladder by increasing CT numbers and reducing noise, as illustrated in Fig. 3 (*p* < 0.001). O-MAR also had a positive effect on noise measured in muscle and fat, which resulted in higher CNRs on O-MAR images compared with no O-MAR images, as CT numbers remained constant. Quantitative results with respect to metal artefact reduction are in concordance with a previous phantom study [13] and other patient and phantom studies, also focusing on the CT imaging of THAs [9, 10, 14–17]. Despite the fact that O-MAR improves image quality by reducing metal artefacts, we recommend additionally evaluating conventional CT images without the use of MAR software, because MAR algorithms may introduce secondary artefacts and could degrade the depiction of bone trabeculae and bone cortex [11, 12, 18–22].

**Fig. 7:**
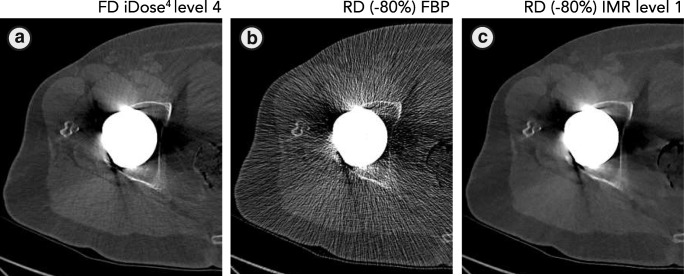
**a** FD iDose^4^ level 4, **b** −80% RD filtered back-projection (FBP) and **c** −80% RD IMR images. With FBP, amplified image noise and artefacts severely deteriorate image quality, whereas IMR is capable of handling the noise and artefacts with comparable image quality to FD iDose^4^ level 4 images

**Fig. 8:**
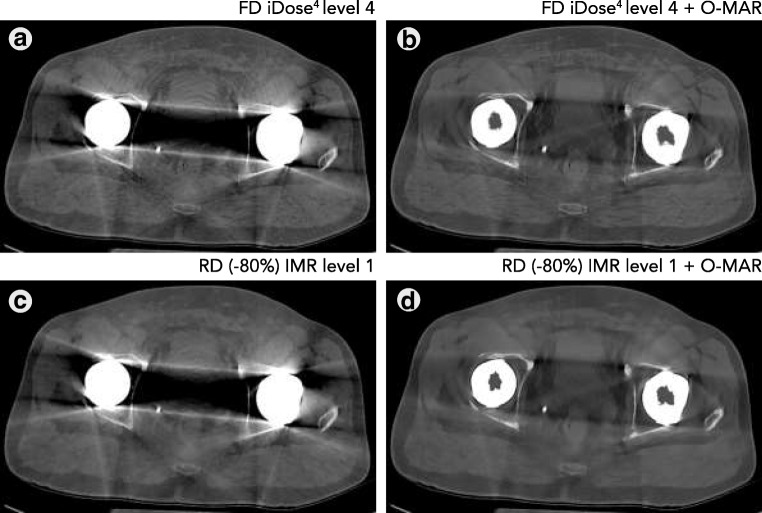
In this case with bilateral metal-on-metal THAs, severe artefacts with extensive photon starvation, are effectively reduced by O-MAR. Image quality of RD (−80%) is inferior to that of FD iDose^4^ images, on both non-MAR and O-MAR images

Filtered back-projection is a fast and robust reconstruction technique, but it cannot deal with artefacts and noise when reducing radiation dose (Fig. 7). We addressed noise as the SD of pixel intensities within an ROI, and are aware that both noise and artefact affect the SD. However, in a previous study we showed that noise reduction by O-MAR is mainly caused by a reduction of metal artefacts resulting in a lower SD, as O-MAR has no influence on images without any metal artefacts [9]. As CT numbers remained similar and noise decreased, CNR improved in RD IMR reconstructions compared with FD iDose^4^ reconstructions with and without the use of O-MAR (Fig. 5). As described in a previous phantom study, lower noise values and higher CNR values are found on RD images compared with FD images, even on −80% RD images [13]. Boudabbous et al. and Kuya et al. found that MBIR furthermore reduces the size of artefacts, and that MBIR allows equal or better visibility of the bone–metal interface and improved assessment of the soft tissue surrounding implants compared with FBP [4, 5]. Yasaka et al. also found that streak artefacts were reduced using MBIR [6]. In contrast to these findings, no distinct metal artefact reduction was observed when using IMR (Figs. 7, 8).

Mean Likert scores (rated from 0 to 3), with respect to artefact and diagnostic quality of 1.59 and 1.56 were found for observers 1 and 2 respectively. In general, image quality was inferior in RD IMR results compared with FD iDose^4^ results. Low Kappa values of 0.16 and lower indicated poor or no agreement between the two observers in RD and FD images. Differences in subjective image quality scoring are not uncommon as there will be a variation in the perception of image quality and viewing strategy among observers, which was also found by Kataria et al. [23]. Despite the fact that there were differences in subjective image quality scores between observers, it is more important to focus on differences between RD and FD scores within observers. For observer 1, the image quality of RD IMR images was rated as inferior to FD iDose^4^ images with respect to: artefacts and diagnostic quality (*p* < 0.001), delineation of bony structures (*p* < 0.001), evaluation of bone density, destruction and sclerosis (*p* < 0.001) and the recto-uterine pouch, the vesico-uterine pouch and the bladder wall (*p* < 0.01; Fig. 6). Similar image quality scores were seen in FD and RD results regarding the evaluation of prosthetic components, pseudo-tumours and the evaluation of relevant hip musculature. Regarding observer 2, the quality of RD IMR images was rated as inferior compared with FD iDose^4^ images on all seven aspects (Fig. 6). Image quality scores were statistically different between the two observers regarding aspects 3, 5 and 7 on FD iDose^4^ images and aspects 5 and 6 on RD IMR images. In most of these aspects, image quality scores of observer 2 were lower compared with observer 1. This can be explained by the fact that observer 2 was less experienced in evaluating IMR images. Second, observer 1 is more experienced in evaluating metal-on-metal THAs and pseudo-tumours.

When focusing on the differences in image quality scores between RD IMR results and FD iDose^4^ results, agreement among observers improved. In most cases, image quality of FD and RD images was rated equal for observer 1 (64.7%) and observer 2 (61.5%). In 25.9% and 30.5% of the cases the image quality of the RD scan was rated 1 point lower compared with the FD scan for observers 1 and 2 respectively. In only 1.7% and 0.9% of the cases was the RD image quality rated 2 points lower compared with the FD scan for observers 1 and 2 respectively. Higher image quality scores were seen in 7.7% and 7.1% of the cases for observers 1 and 2 respectively.

Image quality of RD images reconstructed with IMR is rated lower than images reconstructed with iDose^4^, regardless of the amount of radiation dose reduction. Nevertheless, it can easily be observed that image quality is insufficient in RD IMR results at 80% reduced radiation dose, especially in bilateral metal-on-metal THAs (Fig. 8). The combination of IMR and O-MAR results in a blurring or smoothening effect, especially at the level of the large head of the THA. When there is no or little metal involved, for example in the stem of the prosthesis, this effect was not observed (Figs. 10, 11). When dealing with bilateral metal-on-metal THAs, the use of IMR combined with aggressive radiation dose reduction is therefore discouraged.

We acknowledge that commonly used metrics of image quality, noise and CNR have limited utility in fully assessing image quality for IR and MBIR algorithms [24–26]. Samei et al. suggested that MBIR can potentially make better use of projection data to reduce CT dose by approximately a factor of 2 and furthermore found that MBIR shows improved spatial resolution for high-contrast tasks, but reduced performance for low-contrast tasks at an RD, which may influence low-contrast object detectability [25]. They showed that MBIR has a dose reduction potential of 46–84% compared with ASIR 50%, which is comparable with iDose^4^. MBIR techniques improve spatial resolution while reducing noise at the same time [1–3, 24, 27–29]. We quantitatively assessed CT image quality by measuring CT numbers, noise and CNR, but did not assess spatial resolution. Millon et al. assessed spatial resolution, low-contrast detectability, noise and CNR in a Catphan phantom and chest cadaver study using the same 256-slice iCT scanner and MBIR technique [28]. By determining edge-spread functions, the modulation transfer function, which is a measure of spatial resolution, was calculated. They found that MBIR improved the low-contrast detectability and enabled a CT radiation dose reduction. At an RD, spatial resolution became dose- and contrast-dependent. With an 80% radiation dose reduction, an increasing loss of structures on IMR images was observed and small low-contrast structures started to disappear, which was the case for both FBP and IMR [28]. Therefore, when applying large dose reductions, diagnostic performance could be compromised.

In our opinion, radiation dose can be reduced in THA imaging using MBIR combined with O-MAR, depending on the diagnostic purpose. Especially when evaluating the correct placement and status of prosthetic components in situ, RD protocols are sufficient. We have to keep in mind that image quality is already impaired when imaging a large-head MoM prosthesis; thus, it is unrealistic to expect high image quality scores anyway, especially at the height of the prosthetic head. Despite the fact that image quality was lower in RD IMR results, most soft-tissue abnormalities can be assessed, albeit with reduced diagnostic confidence. Mixed results were found regarding the use of MBIR techniques in soft tissues by other studies. Vardhanabhuti et al., Pooler et al. and Padole et al. reported that small lesions could be missed on RD images using IR and MBIR. Diagnostic confidence and reader confidence for detecting low-contrast lesions were reduced in RD MBIR results compared with the standard dose [7, 30, 31]. In high-contrast regions, a greater reduction of CT radiation dose may be applicable [30].

In a phantom study, Subhas et al. investigated lesion detectability near hardware using MBIR combined with MAR software versus FBP at reduced radiation dose levels and found that a 50% CT radiation dose reduction did not compromise the accuracy compared with FBP. They found that MBIR with MAR was also significantly more sensitive than FBP in detecting smaller lesions and lesions near large amounts of metal artefact [32]. Kataria et al. found that a radiation dose reduction can be applied; however, the diagnostic confidence in evaluating low-contrast objects and details in the liver was impaired using MBIR [23]. It is difficult to compare the outcomes of these studies with our own findings as the number of patients with abnormalities was limited in this population. With respect to evaluating the delineation of bony structures and assessment of bone density, bone destruction and sclerosis, the use of FD iDose^4^ reconstructions is advised. High-contrast details disappear, which could result in the loss of small details and structures such as fractures.

**Fig. 9:**
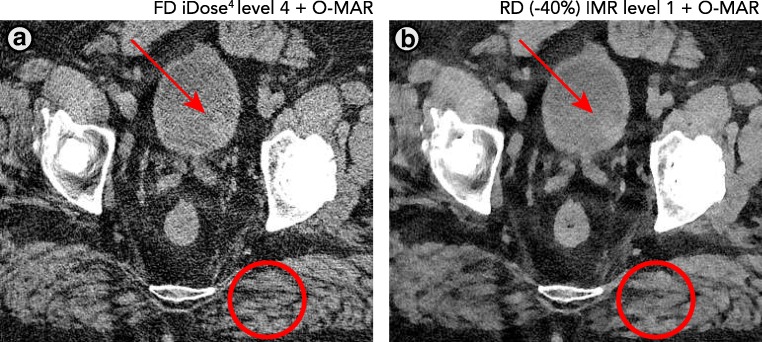
A bladder tumour (indicated by the *red arrow*) can be observed in both the FD iDose^4^ and RD −40% IMR results. Some loss of small details (indicated in the *red circular region of interest*) can be observed when focussing on the delineation of inter-muscular fat

**Fig. 10:**
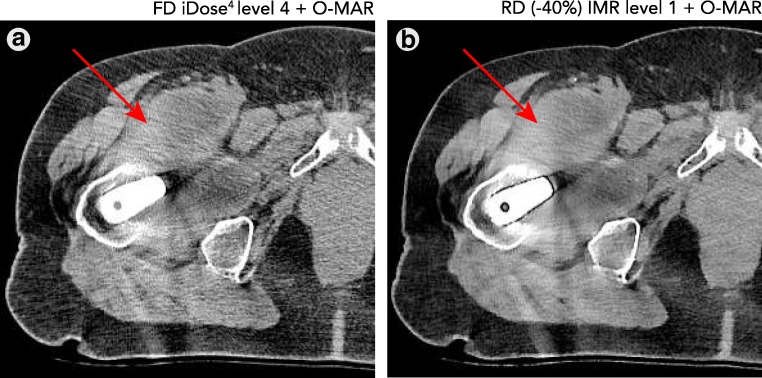
Large pseudo-tumour formation (indicated by the *red arrow*) is observed in both the FD iDose^4^ and RD −40% IMR results

**Fig. 11:**
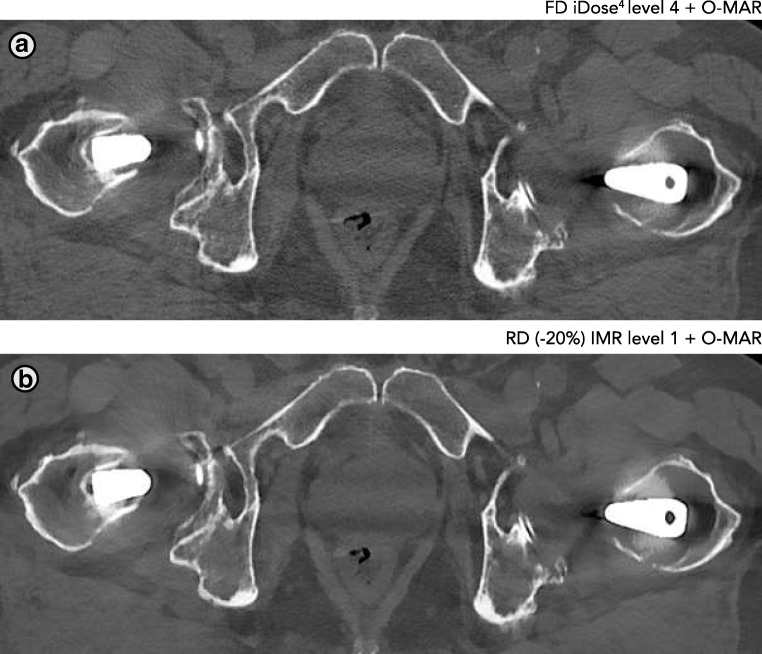
Similar image quality regarding the delineation of bony structures can be observed at the stem of the prosthesis. However, both observers rated the image quality of RD IMR results as inferior to FD iDose^4^ results at the height of the head of the prosthesis

This study has limitations. Observers were blinded to iDose^4^ and IMR results, but one could easily identify IMR images owing to their plastic appearance, which was also encountered in other studies [7, 23]. An extensive training session for both observers could have enhanced the agreement between the observers. Radiologists are used to evaluating images containing noise and adaptation time is required to get used to MBIR images. Additionally, one of the observers was more familiar with evaluating MBIR images, with evaluating metal-on-metal THAs and with the assessment of pseudo-tumour formation. As this study involves follow-up CT scans of patients with metal-on-metal THAs, a relatively low incidence of soft tissue and bone abnormalities was observed. It is therefore hard to determine if clinically relevant findings would have been missed. Figures 9–11 illustrate that findings are also assessed in RD IMR results. It furthermore needs to be noted that the results of this study are generated with the specific IMR reconstruction algorithm using a 256-slice iCT scanner. Results cannot be compared 1-on-1 with MBIR techniques or scanner hardware of other vendors.

In conclusion, CT numbers remained constant, noise decreased and CNRs between muscle and fat increased in RD IMR reconstructions compared with FD iDose^4^ reconstructions for all levels of IMR while reducing CT radiation dose by 20%, 40%, 57% and 80%. Subjective image quality of RD IMR with O-MAR images decreased compared with FD iDose^4^ with O-MAR images in patients with large metal-on-metal THAs, regardless of the degree of radiation dose reduction. Care should be taken when using IMR in combination with reduced CT radiation dose in patients with THAs, especially in the evaluation of osseous structures and when focussing on small details and structures.
